# Wearable Sensors Assess the Effects of Human–Robot Collaboration in Simulated Pollination

**DOI:** 10.3390/s24020577

**Published:** 2024-01-17

**Authors:** Mustafa Ozkan Yerebakan, Boyi Hu

**Affiliations:** Department of Industrial and Systems Engineering, University of Florida, Gainesville, FL 32611, USA; mu.yerebakan@ufl.edu

**Keywords:** human robot collaboration, posture, pollination, agriculture

## Abstract

Pollination for indoor agriculture is hampered by environmental conditions, requiring farmers to pollinate manually. This increases the musculoskeletal illness risk of workers. A potential solution involves Human–Robot Collaboration (HRC) using wearable sensor-based human motion tracking. However, the physical and biomechanical aspects of human interaction with an advanced and intelligent collaborative robot (cobot) during pollination remain unknown. This study explores the impact of HRC on upper body joint angles during pollination tasks and plant height. HRC generally resulted in a significant reduction in joint angles with flexion decreasing by an average of 32.6 degrees (*p* ≤ 0.001) for both shoulders and 30.5 degrees (*p* ≤ 0.001) for the elbows. In addition, shoulder rotation decreased by an average of 19.1 (*p* ≤ 0.001) degrees. However, HRC increased the left elbow supination by 28.3 degrees (*p* ≤ 0.001). The positive effects of HRC were reversed when the robot was unreliable (i.e., missed its target), but this effect was not applicable for the left elbow. The effect of plant height was limited with higher plant height increasing right shoulder rotation but decreasing right elbow pronation. These findings aim to shed light on both the benefits and challenges of HRC in agriculture, providing valuable insights before deploying cobots in indoor agricultural settings.

## 1. Introduction

Farming practices are gradually shifting toward confined spaces, such as greenhouses and high tunnels. Protected agriculture, the type of agriculture that takes place in indoor environments, offers several advantages compared to open-field agriculture, such as protection from harsh climates and adverse weather conditions. However, protected agriculture does have significant shortcomings, particularly in terms of pollination. The hot and humid environment inside affects the behavior of natural pollinators, making them lethargic and, in extreme cases, unable to fly [1]. Additionally, the population of pollinators like bees is decreasing due to various factors, including climate change [2] and the occurrence of diseases like “Isle of Wight” [3]. Researchers have proposed robot-assisted pollination as a supplementary solution to this shortage, with testing being conducted on kiwi farms, Bramble plants, etc. [4,5,6]. Per outcomes such as pollination success rate, fruit quality and system accuracy, these studies have shown the feasibility of using robots for pollination. However, they also highlight several challenges that affect the successful implementation of pollination robots, including flower occlusion and hard-to-reach flower locations that impact detection and pollination accuracy. Unfortunately, these challenges render the implementation of autonomous pollination robotics economically infeasible in the short term.

In recent years, efforts have been made to address the shortcomings of autonomous robotic operations by designing systems that involve both humans and robots collaboratively executing tasks. Designing a system that leverages the strengths of both robots and humans may be beneficial in reducing the efficiency issues faced by the robot and the potential health concerns faced by agricultural workers who encounter these risks. Preliminary results suggest that this approach has reduced flexion angles in other occupational environments [7,8], and has resulted in increased overall accuracy and success rates [9]. The nature of the collaboration varies across industries and specific tasks, but a common feature is that the robot and the human are in close proximity without any physical barriers. This presence of the robot will inevitably affect the posture of the worker, potentially introducing new safety risks. Without knowing whether these changes are helpful or harmful for the worker, implementing such collaborative systems could jeopardize the safety of the workers.

One of the more reliable ways to measure human posture is through wearable sensors. Unlike vision-based systems such as computer vision, which provide highly reliable data but mainly suited for more controlled environments, wearable inertial measurement units (IMUs) can be used in many environments without a substantial loss of accuracy. IMUs have been used in several human–robot collaborative systems for different domains, such as manufacturing [10] and construction [11]. However, their application in evaluating agricultural collaborative systems has been scarce. Therefore, the main purpose of this study is to investigate the effects of human–robot collaboration (HRC) for a manual pollination task through examining the joint angles of shoulders and elbows using wearable sensors to determine whether HRC can reduce the awkward postures assumed by agricultural workers. The rest of the paper is organized as follows: the related work section introduces existing research on pollination robotics and the biomechanical effects of HRC, while the methods section describes our experimental setup, independent and dependent variables, and analysis methods.

## 2. Related Work

### 2.1. Pollination Robots

One of the earliest works on pollination robots began with the work of Wood et al., who proposed the development of miniature flying robots called RoboBees [12]. Chechetka et al. tested the concept of robot-assisted pollination by attaching horsehair bristles coated with an ionic gel to a miniature drone, enabling it to pick up and transfer pollen from one plant to another [13]. Various non-precision-based methods, such as artificially blowing air, spraying pollen, and soap bubbles, have also been adopted [14]. However, for protected agriculture, space constraints make the implementation of these solutions difficult. Recent advancements in robotic perception, planning, control, and autonomy have enabled researchers to implement precision-contact-based pollination [5] that mimics the actions of natural pollinators. In this study, the robotic pollinating system achieved a detection accuracy of 93.1% and a pollination success rate of 76.9%. While these accuracy and success rates are commendable for the experimental stage, they may not be a viable solution for stakeholders considering investing in such systems or for the agricultural workers who will collaborate or work alongside these robots.

### 2.2. Studies on the Biomechanical Effects of HRC

Using physiological measures to enhance HRC has been a topic of interest for several years. Humans possess various potential “cues”, such as posture, muscle activation levels, and eye fixation, which can inform their collaborative robot partners of their intentions. This information can be utilized to improve the safety and productivity of the collaborative task. Several HRC frameworks have been proposed that exploit these cues to provide workers with assistance in collaborative tasks [15,16,17,18]. For instance, [18] developed an HRC framework that focused on joint torques, muscle activation, and manipulability (defined as the ease of task completion). Depending on the desired outcomes, the robot configurations were able to be adjusted to provide the desired support to the worker. Another model proposed by [17] emphasizes reducing muscle activity. Their model assumes that humans will adopt postures or limb configurations that minimize muscle activity while ensuring the stability of the robot’s joints and grasps. They applied their model to three different tasks: puncturing, pulling, and cutting.

As mentioned, some existing HRC systems rely on biomechanical cues to better inform collaborative robot algorithms. There are several ways to obtain these biomechanical cues. Some studies utilize computer vision algorithms to recognize body postures associated with different activities, including picking [19,20] and lifting tasks in agriculture. Although these computer vision algorithms are fairly accurate, vision-based activity recognition methods, such as computer vision, frequently suffer from occlusion-related accuracy issues [21]. Hence, in unstructured and semi-structured environments like greenhouses, solely relying on vision-based methods to develop HRC systems would not be efficient. In addition to the accuracy issues, there are important outcomes such as muscle activation and brain activity that vision-based systems simply cannot measure. As an alternative, other studies have utilized body-worn sensors. For example, [22] proposed a system that used heart rate, electromyography (EMG), electroencephalogram (EEG), nose temperature, and an inertial measurement unit (IMU) for head movement to communicate human intention. Another proposed system uses IMUs to translate the movements of the upper limbs to control the movements of a robotic manipulator [23].

While there have been studies on the biomechanical effects of HRC using wearable sensors in other fields, similar studies conducted in the agriculture field are quite rare, especially those involving advanced and intelligent collaborative robots (cobots). One study by Benos et al. investigated the appropriate height configuration for a harvesting robot [24]. Their study focused on optimizing the loads on the lower back of agricultural workers as they lifted produce baskets. Although this study suggested an ideal height configuration of 90 cm, the robot was not physically present in their research. Without knowing how the physical presence of the robot influences posture and muscle activation levels, further investigation is needed. Therefore, there is a need for studies in the agriculture field that incorporate HRC scenarios similar to those conducted in industrial/manufacturing applications.

## 3. Methods

### 3.1. Participants

A total of 16 participants (11 males, 5 females) were recruited for the experiment. The average age of the participants was 28.06 (±7.01) years, and the average height was 175.3 cm (±11.8 cm). The sample size of this study is comparable to that of other studies conducted in the field of HRC [25]. There were two inclusion criteria and one exclusion criterion for participation. The inclusion criteria specified that participants should be at least 18 years old and have either normal vision or corrected-to-normal vision. Individuals with a history of musculoskeletal diseases were excluded from the study.

### 3.2. Bioinstrumentation

**Figure 1:**
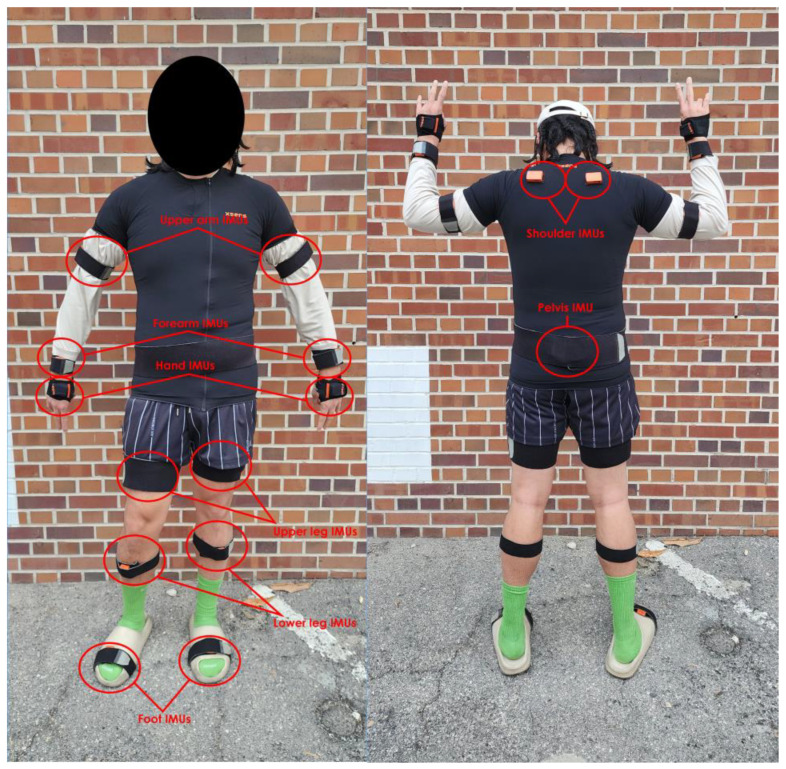
The full-body Xsens Awinda IMU system setup.

To record the kinematics data, the Xsens Awinda IMU system (Movella, Enschede, The Netherlands) was used with the full body set of 17 IMU sensors being attached to the head, sternum, pelvis, right and left hand, right and left shoulder, right and left upper arm and forearm, right and left upper and lower leg, and right and left foot (Figure 1). Each of the IMUs consists of an accelerometer, magnetometer, gyroscope, and barometer; however, for this study, only the accelerometer data were used. Before data acquisition, body measurements of the participants were taken to ensure the sensors were calibrated accordingly. Calibration was only completed once before the trials started and only repeated if the IMU system malfunctioned. The data that were collected were the joint angle values for the right and left shoulders and elbows. 

### 3.3. Experiment Setup and Task Description

**Figure 2:**
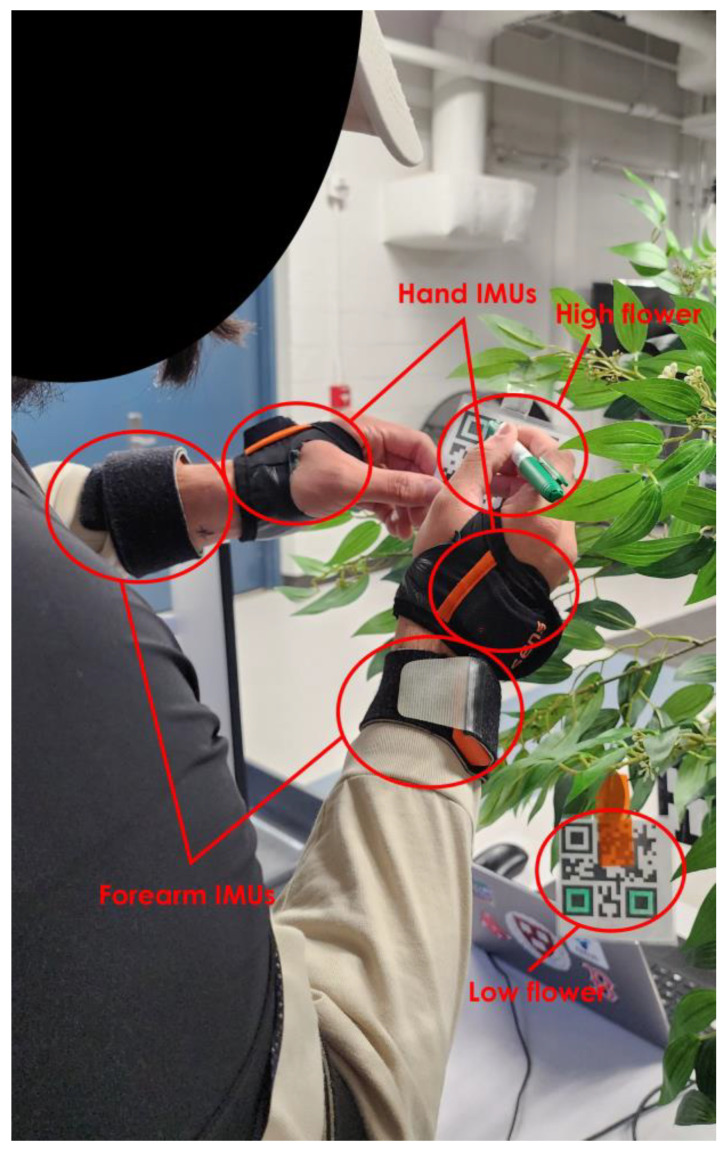
An example of the simulated pollination task setup. The participant is coloring the top or bottom adjacent two squares on the QR codes. Orange tape and red numbers indicate low height condition, and clear tape with black numbers indicates high height condition.

To simulate robotic pollination, a Universal Robotics-5 (Odense, Denmark) collaborative robot was used. An artificial plant was employed for the simulated pollination task (Figure 2). The plant was positioned on top of a desk at a fixed height. Participants stood approximately 60 cm away from the midpoint of the plant pot, as indicated by a fixed position marked on the ground. The total height of the plant with the table was 168 cm. The pollination task was approximated by using QR codes (referred to as “flowers” in this manuscript), and the pollination task was mimicked through a hand-coloring operation for two primary reasons: (1) natural plants have limited bloom periods, which would restrict the study’s timeframe, and (2) using actual flowers would have made it challenging to ensure that participants conducted pollination consistently (Figure 2). This approach is based on previous research that used QR codes as representations of flowers in a pollinator robot experiment [4]. 

In total, there were 20 simulated flowers distributed across the plant, with 10 placed in the upper half and 10 in the bottom half. These flowers were numbered from 1 to 10 for both the upper and lower halves. To clearly identify which flowers were considered “high” and “low,” different colored tapes were used: orange for low and clear for high (Figure 2). Participants were informed of this distinction before the trials began. The numbers written on the flowers were red for the “low” flowers and black for the “high” flowers. Distinguishing the flowers in this way ensured that the participants did not make mistakes while conducting specific experimental conditions. Enumerating the flowers helped guide the participants during the collaborative conditions with the robot. The average height for the “low” flowers was 26 cm (measured from the table), and for the “high” flowers, it was 59 cm. There were a total of 8 different conditions in the experiment (i.e., 2 heights × 4 HRC modes), each repeated twice, resulting in 16 trials for each participant. For the HRC trials, during the experiment, the participants and robot started their respective tasks simultaneously. If the participant finished their task earlier than the robot, they would wait until the robot also finished its task before starting another simulated pollination operation. This protocol was established for the safety of the participants and to ensure that the participant and robot worked simultaneously for each flower.

**Figure 3:**
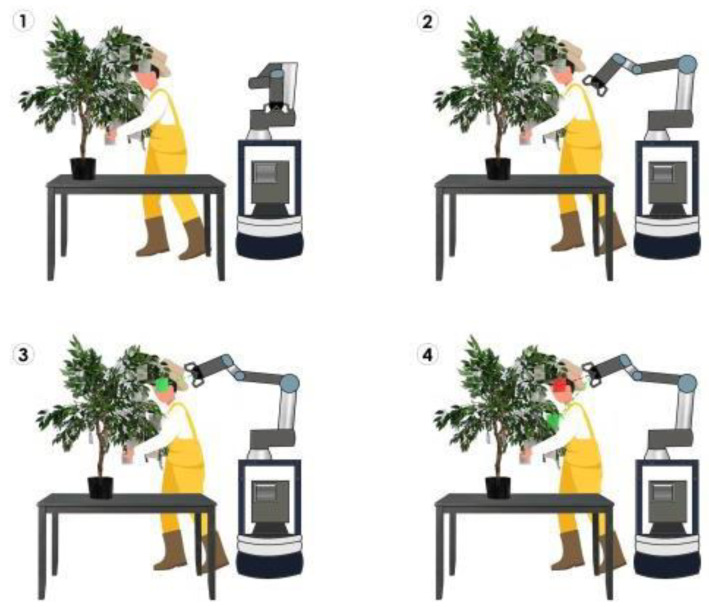
The HRC modes that were evaluated in this study. (**1**) Control with no robot or “manual”, (**2**) unknown mode (no targets indicating the participant did not know the robot’s intended target), (**3**) known mode (the green target and dashed line indicating the robot’s target being known by the participant) (**4**) unreliable mode (the green indicating the robot’s intended target and red target and dashed line indicated the robot made a mistake).

There were two independent variables tested in this experiment: the height of the plant and the HRC configurations. The height of the plant had two levels: high and low. The HRC configurations had four levels: no HRC, “unknown” (participants were unaware of the robot’s target flower), “known” (participants were informed about the robot’s target flower), and “unreliable” (the robot would miss two of its target flowers) (Figure 3).

As mentioned earlier, the 20 flowers were divided into 10 high and 10 low categories to represent the varying heights of plants typically found in greenhouses, ranging from shorter plants like cucumbers and blackberries to taller plants like tomatoes. The HRC modes symbolize different stages of development for a potential HRC system in a pollination robot. The “unknown” condition represents the basic level of HRC, where the participant lacks directional and intentional cues from the robot. In the “known” level, these cues are present. In this experiment, the experimenter verbally communicated the target location and additionally indicated it by touching the point before the trials began. The “unreliable” condition simulated potential accuracy issues that robots might face in detecting and articulating to a pollination plant. While image recognition algorithms often report accuracy rates of 85–95% [26], these studies typically use still images that do not fully represent real-world scenarios. In reality, flowers are frequently occluded by leaves or located within the inner parts of plants, making such high accuracy rates unfeasible. Thus, we adopted a more conservative estimate of 60%, which aligns with the accuracy rates observed in other agricultural robot studies conducted in the field. The primary category of variables included in this study was the upper extremity kinematics, which encompassed the abduction/adduction, rotation, and flexion/extension angles for the shoulders and elbows on both sides.

### 3.4. Protocol

After obtaining the participants’ consent, they were first provided with an explanation of the task and the experimental conditions. Following this, demographic information, including age and gender, was collected from the participants. Once the questionnaire was completed, the participants proceeded to put on the IMU sensors. In the manual condition, participants worked on all 10 flowers within a specific height category individually. In the HRC trials, the flowers were evenly divided between the robot and the participant, with the flowers closer to the participant being assigned to them and vice versa for the cobot. For each HRC trial, participants were assigned a predetermined order of QR codes to color. To maintain consistent movements across all HRC conditions, only the order of the points was altered, while the numbers remained the same for their respective assigned heights. The robot also had a specific order of flowers it needed to touch, which remained consistent to prioritize safety and minimize variations in movement. These movements were pre-programmed using the dedicated software of UR-5, and the pollination interaction was simulated as the end effector clasps closing and touching the QR code. The cobot then assumed its original position for the next flower. After completing each condition, participants were allowed to take a short break to mitigate the effects of fatigue.

### 3.5. Data Processing and Statistical Analysis

The kinematics data were processed by the built-in software that was part of the Awinda IMU data collection software. The sampling frequency of which the data was recorded was 1000 Hz with a 184 Hz low-pass filter applied afterwards [27]. The video output of the participants’ movements during the trials was captured at a 60 Hz framerate. After data processing was complete, the human kinematics data were exported to Microsoft Excel for further analysis. The final kinematics values for each experiment condition were calculated by obtaining the average value of each trial and taking the average of the two repetitions that would make an experiment condition.

Before conducting any formal statistical analysis, the dataset was assessed for normality using the Kolmogorov–Smirnov test [28] to verify the assumption. For the kinematics data that met the assumption of normality, two-way Repeated Measures ANOVA (RM-ANOVA) analyses were performed [29]. To test if the data violated the assumption of sphericity, Mauchly’s test was applied. In cases where the assumption was violated, necessary corrections using the Greenhouse–Geisser or Huynh–Feldt epsilon values were applied [30]. Once the main effects were determined to be significant, the simple main effects of height and robot mode were individually assessed using post hoc pairwise comparisons with Bonferroni corrections applied.

## 4. Results

The effect of flower height did not significantly influence upper arm kinematics, except for right shoulder rotation (Table 1), where a higher flower height induced a joint angle increase of 5.2 degrees (Table 2). However, the effect of robot mode was much more pronounced. The introduction of the robot resulted in reduced rotation and flexion for both shoulders. Specifically, shoulder rotation was reduced by about 20 degrees between the manual and known conditions for both shoulders, and flexion was reduced by 32.5 degrees. The reduction in the flexion angle was not sustained when unreliability was introduced. For the left and right shoulders, the unreliable condition resulted in an increase of 9.2 and 11.5 degrees. Unreliability did not have the same effect on the shoulder rotation values with any observed increases being insignificant.

In terms of the elbows, the robot mode had a significant effect on the flexion angle for the right elbow and the rotation and flexion angle for the left elbow (Table 3). For the right elbow, the known condition reduced the flexion angle by 29.5 degrees compared to the manual condition (Table 4). For the left elbow, the effect of the known condition was opposite for the flexion and rotation parameters. The known condition increased the rotation by 18.75 degrees, whereas the flexion angle decreased by 21.5 degrees, similar to the right elbow.

## 5. Discussion

The purpose of this study was to explore the biomechanical effects of working with a cobot during a simulated agricultural pollination task using a wearable IMU system. Specifically, we were interested in determining whether collaborating with a robot would lead to a reduction in awkward postures by causing a decrease in joint angles. As manual pollination is a task that primarily involves the upper limbs, we collected kinematics data for the shoulder and elbow joints on both sides to analyze the effects of HRC. Wearable IMUs were chosen to evaluate the kinematics data, as their feasibility has been verified by previous studies for upper limb kinematics [31].

The independent variables included in the study represent two different aspects of pollination relevant to a collaborative task. Plant height is crucial because greenhouse-cultivated plants vary significantly in height. For instance, tomato plants have been shown to range from 150 cm to nearly 350 cm for the tallest specimens [32], while blackberries, another common greenhouse fruit, can reach heights ranging from 7 to 15 feet [33]. Additionally, in some indoor agricultural scenarios, it is typical to place plants on top of tables or benches to ensure more ergonomic working postures for growers, further emphasizing the need to accurately represent this variety in the simulated pollination task.

The effect of height was observed primarily on the right-hand side of the participants with higher plant height inducing higher shoulder rotation (*p* = 0.005) (Table 1) and elbow pronation (*p* = 0.028) (Table 3). The increased shoulder rotation, along with the slight increase in abduction for lower plant heights, might be attributed to the hunched position assumed by participants. As they lowered their trunks to reach flowers, they may have compensated for their posture by rotating their shoulders more to orient themselves. However, with taller flowers, participants maintained a straighter posture, reducing the need for compensatory adjustments.

The second independent variable, the inclusion of different robot modes, represents the different stages of prototype development of a pollination robot. The unknown condition represents the base functionality of the robot. The known condition is a potential addition to the robot’s capabilities to enhance the situational awareness of the human collaborator. The “unreliable” condition represents the most realistic version of the design with possible failures being simulated through the cobot missing the target and the human collaborator monitoring to compensate for the cobot’s mistake. Human supervision of robots during collaborative tasks has been observed in repetitive industrial tasks [34], and although the pollination task and its setting are different from manufacturing, it is a repetitive task with flower location being the main variable. Hence, the “unreliable” condition was simulated as a collaborative task with human supervision.

**Figure 4:**
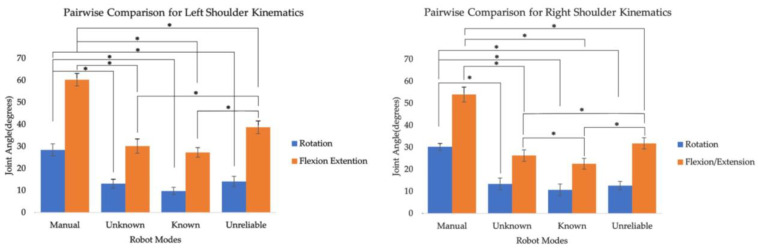
Pairwise comparison for shoulder kinematics with significant effects for robot mode. The * indicates significant difference between the conditions.

The impact of the robot mode varied across different movement planes with HRC modes reducing joint angles in the transverse and sagittal planes (*p* ≤ 0.001) for both shoulders (Figure 4) and in the sagittal plane for both elbows (*p* ≤ 0.001) while increasing rotation for the left elbow (*p* ≤ 0.001). The decreased rotation and flexion for both shoulders likely resulted from task allocation between the cobot and the human worker. In the manual condition, the human worker had to pollinate all ten flowers, which were placed at varying distances. As participants reached further flowers, plant branches caused more occlusion, necessitating more awkward postures. However, when tasks were evenly divided between the cobot and the human worker, the human worker only pollinated nearby flowers, leaving the farther ones for the cobot. This result underscores the importance of designing tasks to align with environmental demands and the capabilities of collaborative agents, which is consistent with previous studies [35,36].

**Figure 5:**
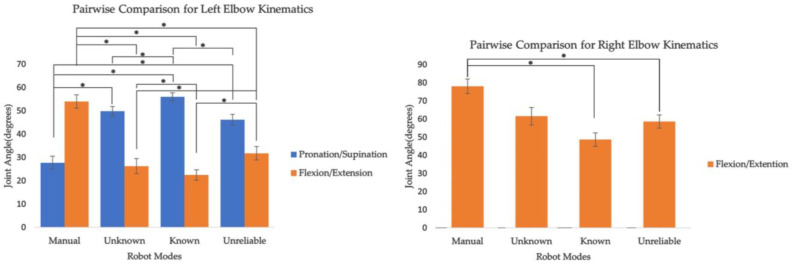
Pairwise comparison for elbow kinematics with significant effects for robot mode. The * indicates significant difference between the conditions.

Similar to the improvements observed for the shoulders, it can be argued that the reduction in joint angles in the sagittal plane for both elbows and the reduction in the right elbow’s transverse plane can be attributed to task allocation between the cobot and the human worker, which reduces the awkward postures associated with more distant flowers (Figure 5). However, the increased pronation observed for the left elbow when the cobot was introduced indicates a different behavior. It can be argued that the presence of the robot may prompt workers to align flowers closer to themselves by rotating them.

**Figure 6:**
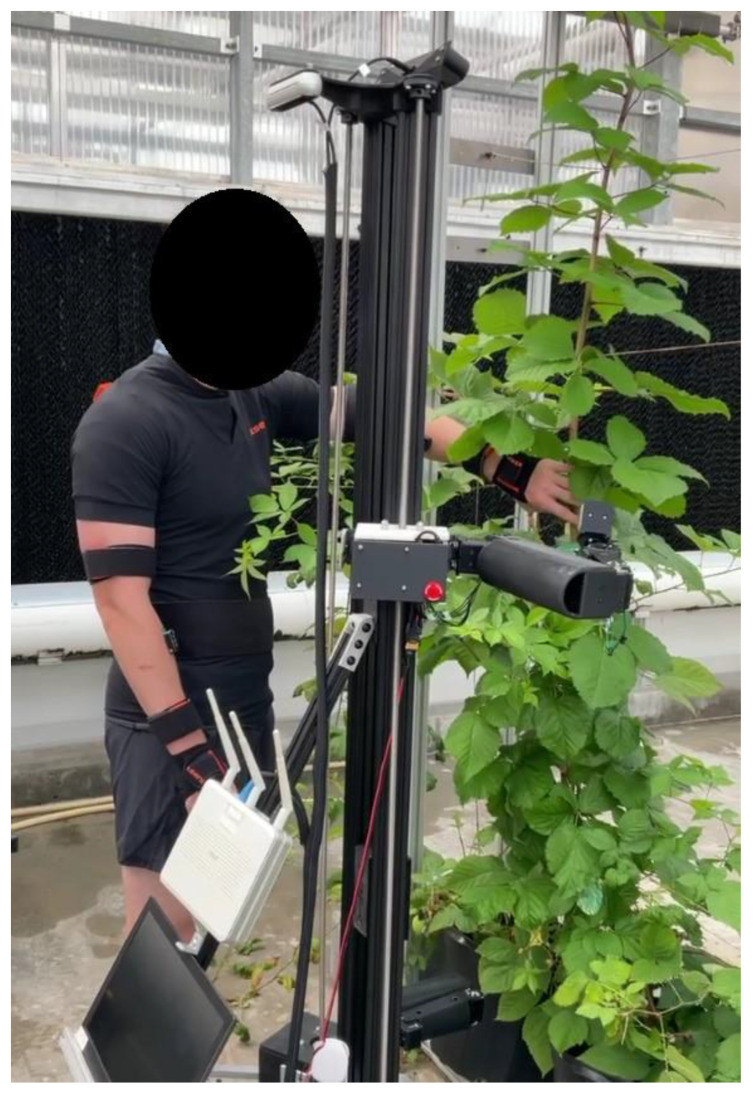
Future collaborative disease detection application using wearable IMU.

This study has notable limitations that need to be acknowledged. Firstly, the omission of gender as an independent variable restricts the generalizability of the results. Research has shown gender-based differences among agricultural workers with females experiencing more musculoskeletal discomfort than males [37]. Additionally, on average, females are shorter than males, which can influence assumed postures. Another limitation is the study’s narrow focus on the upper body kinematics of the participants. A more comprehensive understanding of the effects of plant height and HRC can be obtained by including spine biomechanics, electromyography, and subjective measures such as NASA-TLX. Future studies will address these considerations and ensure that the experimental environment is more representative of indoor agriculture. An example of ongoing research efforts can be seen in Figure 6 for a collaborative disease detection task. Additionally, efforts will be made to recruit participants who are more representative of the target population for collaborative pollination applications. Another potential avenue is to investigate behavioral differences across various application areas of agricultural robotics and whether these differences are impacted by interacting with or observing the robotic applications firsthand, given the diverse nature of tasks and varying levels of acceptance among agricultural stakeholders [38].

## 6. Conclusions

The initial hypotheses regarding the low plant condition and non-HRC condition inducing more awkward postures were partially supported. Higher plant conditions were found to induce greater rotation for the right shoulder, while the effect on pronation/supination for the left elbow was the opposite. The influence of HRC was evident for most movement planes across all included body segments. In general, HRC led to decreased joint angles compared to the manual condition, except for the left elbow pronation/supination, where the “known” robot mode increased pronation. The positive effects of HRC were reversed when unreliability was introduced. However, the left elbow pronation/supination remained an exception to this trend. The findings of this study may underscore the complex effects and potential benefits of HRC in the agricultural context. Moreover, the study highlights the challenges that must be considered before cobots are deployed more ubiquitously.

## Figures and Tables

**Table 1:** RM-ANOVA results for the shoulder kinematics (*p*-values).

Independent Variable	Right Shoulder			Left Shoulder		
	Ab/Add	Rotation	Flex/Ex	Ab/Add	Rotation	Flex/Ex
Height	0.204	**0.005**	0.131	0.788	0.508	0.963
Robot Mode	**0.017**	**<0.001**	**<0.001**	**0.021**	**<0.001**	**<0.001**
Height*Robot Mode	**0.008**	0.625	0.188	0.248	0.200	0.667

Bold numbers indicate significant difference (*p* ≤ 0.05); Ab/Add: Abduction/Adduction, Flex/Ex: Flexion/Extension. * indicates the interaction effect between the independent variables.

**Table 2:** Mean and (standard deviation) of the shoulder joint angles (in degrees).

Robot Mode	Right Shoulder			Left Shoulder		
	Abb/Add	Rotation	Flex/Ex	Abb/Add	Rotation	Flex/Ex
Manual	3.95 (2.47)	**30.15 (1.59) ^A^**	**54.00 (3.37) ^A^**	4.90 (2.19)	**28.39 (2.77) ^A^**	**60.28 (2.87) ^A^**
Unknown	9.27 (1.27)	**13.36 (2.66) ^B^**	**26.25 (2.59) ^C^**	8.81 (1.83)	**13.07 (2.05) ^B^**	**30.18 (3.24) ^C^**
Known	9.40 (1.10)	**10.70 (2.62) ^B^**	**22.50 (2.45) ^D^**	9.44 (1.29)	**9.72 (1.67) ^B^**	**27.27 (2.20) ^C^**
Unreliable	9.35 (1.34)	**12.63 (1.89) ^B^**	**31.77 (2.52) ^B^**	8.86 (1.13)	**14.08 (2.31) ^B^**	**38.71 (2.89) ^B^**
**Height**						
Low	8.79 (1.25)	**14.11 (2.11)**	31.40 (3.17)	7.86 (1.47)	16.63 (1.95)	39.16 (2.83)
High	7.20 (1.60)	**19.31 (2.06)**	35.86 (2.65)	8.14 (1.45)	15.99 (1.63)	39.05 (2.74)

Different numbers in the superscript indicate significant difference (*p* ≤ 0.05).

**Table 3:** RM-ANOVA results for the elbow kinematics (*p*-values).

Independent Variable	Right Elbow			Left Elbow		
	Abb/Add	Pro/Sup	Flex/Ex	Abb/Add	Pro/Supp	Flex/Ex
Height	0.901	**0.028**	0.873	0.097	0.658	0.127
Robot Mode	0.042	0.658	**<0.001**	0.745	**<0.001**	**<0.001**
Height*Robot Mode	0.805	0.235	0.351	0.772	0.594	0.140

Bold numbers indicate significant difference (*p* ≤ 0.05); Ab/Add: Abduction/Adduction, Pro/Sup: Pronation/Supination, Flex/Ex: Flexion/Extension. * indicates the interaction effect between the independent variables.

**Table 4:** Mean and (standard deviation) of the elbow joint angles (in degrees).

Robot Mode	Right Elbow			Left Elbow		
	Abb/Add	Pro/Supp	Flex/Ex	Abb/Add	Pro/Supp	Flex/Ex
Manual	−4.66 (4.00)	71.46 (5.93)	**78.10 (4.02) ^A^**	−24.66 (2.69)	**27.75 (3.89) ^C^**	**54.00 (3.37) ^A^**
Unknown	−12.99 (3.28)	74.59 (4.65)	**61.56 (4.82) ^AB^**	−24.70 (2.04)	**49.81 (3.28) ^B^**	**26.25 (2.59) ^C^**
Known	−11.73 (2.58)	75.46 (6.45)	**48.68 (3.70) ^B^**	−23.27 (1.82)	**56.03 (3.32) ^A^**	**22.50 (2.46) ^D^**
Unreliable	−6.66 (2.84)	76.52 (4.98)	**58.63 (3.62) ^B^**	−23.75 (1.72)	**46.16 (3.18) ^B^**	**31.77 (2.52) ^B^**
**Height**						
Low	−9.18 (3.65)	**78.01 (4.78)**	61.37 (3.24)	−25.40 (1.99)	44.64 (3.21)	31.40 (3.17)
High	−8.85 (2.02)	**71.01 (5.45)**	62.11 (3.37)	−22.79 (1.75)	45.24 (3.20)	35.86 (2.65)

Different numbers in the superscript indicate significant difference (*p* ≤ 0.05).

## Data Availability

Data are contained within the article.
